# The relationship between thanatophobia and perceptions of spirituality and spiritual care among intensive care nurses

**DOI:** 10.3389/fpsyg.2026.1945404

**Published:** 2026-09-23

**Authors:** Hilal Erer, Gülnur Akkaya

**Affiliations:** 1 Mehmet Akif Ersoy State Hospital, Çanakkale, Türkiye; 2 Department of Nursing, Faculty of Health Sciences, Çanakkale Onsekiz Mart University, Çanakkale, Türkiye

**Keywords:** attitude to death, critical care nursing, nurse, spirituality, spiritual therapies

## Abstract

**Aim:**

This study aims to determine the relationship between thanatophobia and perceptions of spirituality and spiritual care among intensive care nurses. The research was conducted using a descriptive and correlational design.

**Methods:**

This study included 198 nurses working in the intensive care units of state and university hospitals in a province in western Turkey between January 2025 and April 2025. Data were collected using three different instruments: a sociodemographic information form, the Thanatophobia Scale (TS), and the Spirituality and Spiritual Care Rating Scale (SSCRS).

**Results:**

The mean TS score of the nurses participating in the study was 26.52 ± 9.02 (high). The mean SSCRS score was 52.79 ± 9.22 (high). A significant positive correlation was found between TS and SSCRS (*r* = 0.28; *p* < 0.001). The effect of TS on SSCRS was positive and significant (β = 0.28, *p* < 0.001). The coefficient of determination of the model (R^2^ = 0.07) indicates that TS explains 7% (medium) of the variation in SSCRS.

**Conclusion:**

This study found that as the fear of death increased among intensive care nurses, their perception of spirituality and spiritual care also increased moderately. Since intensive care nurses frequently encounter death, it is recommended that they receive institutional support to cope with their fear of death. Furthermore, it is suggested that interdisciplinary training programs be organized and provided to enable intensive care nurses to provide spiritual care.

## Introduction

Death, an inevitable part of life, evokes fear in many individuals for various reasons, including the end of life, annihilation, and separation from loved ones. Yalom (1980), one of the pioneers of existential psychotherapy, defines death anxiety as one of the fundamental existential crises that humans must confront. According to Yalom (2008), although death awareness creates feelings of meaninglessness and isolation in the individual, it carries a powerful transformative potential for re-evaluating the meaning of life. One of the most comprehensive approaches in psychology and social sciences to explain death anxiety and its effects on human behavior is Terror Management Theory (TMT) (Greenberg et al., 1986). Based on Becker’s (1973) existentialist studies, this theory suggests that when individuals confront the inevitable reality of mortality, they buffer the existential terror they experience by clinging to their cultural worldview, self-esteem, and spiritual values (Greenberg et al., 1997). The transformation of death anxiety into a spiritual caregiving behavior in nurses working in clinical settings, especially in high-risk areas such as intensive care units, is also explained by this psychological shielding function (Sharif Nia et al., 2020). Thanatophobia may prevent individuals from expressing their emotions about death; it encompasses not only fear of death itself, but also fears related to pain, loneliness, uncertainty, and nonexistence, and perceptions of death vary from person to person (Arab et al., 2019; Üstükuş and Eskimez, 2021; Çamlıca et al., 2024). The idea of death generates a specific form of anxiety referred to as death anxiety (DA). According to the Nursing Outcomes Classification, DA is defined as “a vague and uneasy feeling of discomfort or dread generated by a perceived threat to one’s existence, whether real or imagined” (Sharif Nia et al., 2020). DA is generally conceptualized as a sense of worry, terror, or anxiety triggered by thoughts of death. When considered as a classical phobia, thanatophobia is characterized by the individual’s inability to confront the direct possibility of death, resulting in an abstract fear (Sharif Nia et al., 2020; Qutishat, 2025).

Intensive care units (ICUs) are high-technology, multidisciplinary environments where patients with life-threatening conditions and multiple organ failure receive treatment and care, and where mortality and morbidity rates are high (Chen et al., 2023; Meşe et al., 2025). Intensive care nurses (ICNs) remain with patients 24 h a day, continuously assess them, and provide holistic care. Compared with other healthcare professionals, they engage in more frequent communication and collaboration with patients, family members, and other disciplines (Uçar et al., 2024). ICU nurses encounter death more frequently than nurses working in other death anxiety and death-related fears (Arab et al., 2019; Kartal et al., 2024; Qutishat, 2025). ICNs strive to meet the needs and expectations of dying patients, complete all necessary preparations for transferring the deceased to the morgue, and communicate with family members. Nurses who experience fear of death may feel distress while fulfilling these responsibilities and may develop feelings such as sadness, fatigue, helplessness, burnout, and psychological trauma, which may hinder their ability to provide holistic care and may reduce the quality of care (Arab et al., 2019; Karkhah et al., 2024; Kudubes et al., 2021; Medin et al., 2020; Qutishat, 2025; Semerci et al., 2021; Zulfiqar et al., 2021). Studies on this topic indicate that ICNs spend substantial time with dying patients and experience high levels of death fear [1,5,11,13], (Arab et al., 2019; Zulfiqar et al., 2021; Kudubes et al., 2021; Qutishat, 2025). ICU nurses work in an environment where they are frequently exposed to acute clinical death events (e.g., cardiac arrest, end-of-life crises) rather than abstract discussions of death. Therefore, the assessment of thanatophobia, the acute, reactive fear triggered by direct encounters with death, is considered theoretically more appropriate for capturing immediate psychological coping mechanisms i.e., engaging in spiritual care behaviors as a protective buffer according to TMT rather than broader attitudes towards death or general anxiety.

Spirituality encompasses individuals’ spiritual experiences, beliefs, values, culture, community, conscience, and mental health–related components, and focuses on the meaning, purpose, and values of life. Therefore, spirituality is regarded as a power that nourishes, strengthens, and guides the person’s inner world and soul (Tan and Yıldız, 2022). Challenging situations such as illness, pain, loss, and death may bring spiritual needs to the forefront. Nurses should be sensitive in recognizing these situations and in responding to patients’ spiritual needs (McSherry et al., 2002; Ergül and Bayık, 2007; El-Ashry et al., 2025). The health policy implementation and research guidelines emphasize that spiritual care is an integral component of holistic care and is of great importance for quality of life (Dalcalı et al., 2021; Karacan et al., 2024). Spiritual care is a critical element of high-quality and holistic nursing care and encompasses diverse religious and spiritual beliefs, psychological dimensions, and moral and cultural aspects (Baysal et al., 2024; Wang et al., 2024; Timmins et al., 2025). Providing spiritual care contributes to reducing patients’ helplessness, negative emotions, and even the severity of disease-related symptom (Heidari et al., 2025), while enhancing their spiritual well-being, hope, and life satisfaction (Wang et al., 2024). Spiritual care supports patients in coping with emotional and spiritual distress and aims to improve their overall well-being. Techniques such as religious rituals, meditation, prayer, and counseling may form part of this approach. The goal is to address each patient’s spiritual needs with respect for their personal beliefs and values (Akpınar et al., 2025). When nurses are knowledgeable about the importance of meeting patients’ spiritual and religious needs, they are more likely to provide spiritual care (Hosseini et al., 2022; Anshasi et al., 2024). Nurses who are able to deliver appropriate spiritual care are also more likely to experience spiritual satisfaction (Özakar Akça et al., 2022). However, to identify patients’ spiritual needs, nurses must first have an accurate understanding of spirituality and spiritual care. Higher perceptions of spiritual care among nurses are associated with greater spiritual care competence (Wang et al., 2024; Karaman and Sağkal Midilli, 2022). Spiritual care is a key component of holistic nursing in settings where patients face severe illness or risk of death, including palliative care units, pain clinics, oncology wards, and intensive care units (Umberger and Wilson, 2024).

ICU nurses are routinely exposed to rapid patient deterioration and end-of-life crises. If unaddressed thanatophobia impairs a nurse’s ability to provide spiritual care, patient outcomes and family satisfaction during end-of-life care deteriorate. Conversely, if spiritual care serves as a protective coping strategy for nurses, fostering spiritual competence can mitigate compassion fatigue, moral distress, and burnout. Therefore, establishing this empirical link provides a crucial foundation for developing targeted educational interventions and psychological support systems tailored for critical care staff. In this context, spiritual care provided in a clinical setting functions as a critical therapeutic tool in filling the existential void created by death anxiety and preserving the individual’s meaning system.

In Türkiye, the Nursing Regulation states that nurses are required to approach individuals holistically in fulfilling their roles, authorities, and responsibilities (Regulation Amending the Nursing Regulation, 2011). The International Council of Nurses (ICN) Code of Ethics recommends that nurses “promote an environment in which the religious and spiritual beliefs of the individual, family, and community are respected by all” (International Council of Nursing [ICN], 2021). In 2025, ICN updated the definition of nursing, emphasizing that “nurses enhance health literacy, promote health, prevent illness, protect patient safety, alleviate suffering, facilitate healing and adaptation, and preserve dignity throughout life and at the end of life” (International Council of Nursing [ICN], 2025).

Türkiye is a country where the majority of the population is Muslim and in 2015, through collaboration between the Ministry of Health of the Republic of Türkiye and the Presidency of Religious Affairs, spiritual care units were established in hospitals, and spiritual care personnel were appointed to provide spiritual support to patients, relatives, and healthcare professionals upon request (Guidelines for the Providing of Spiritual Support Services in Health Care Institutions, 2015).

The hypotheses of the study are:

1. Intensive care nurses have a high level of thanatophobia.

2. Intensive care nurses have a high perception of spiritual care.

3. As the level of thanatophobia increases among intensive care nurses, their perception of spiritual care also increases.

## Materials and methods

### Design and reporting

A cross-sectional, descriptive, and correlational design was used. Reporting was guided by the Strengthening the Reporting of Observational Studies in Epidemiology (STROBE) statement for cross-sectional studies (von Elm et al., 2007). The study was observational; no temporal or causal inference was intended.

### Setting and participants

The study was conducted in adult intensive care units of a state hospital and a university hospital in one province in western Türkiye. During the study period, the state hospital had 66 adult intensive care beds and 125 nurses across cardiovascular surgery, neurology, anesthesia and reanimation, coronary, and general intensive care units. The university hospital had 86 adult intensive care beds and 100 nurses across coronary, cardiovascular surgery, anesthesia and reanimation, and general intensive care units.

A census-oriented recruitment approach was used. The accessible workforce comprised 225 nurses listed in the adult intensive care units during the study period. Eligible participants were registered nurses who had worked in an adult intensive care unit for at least 1 year, volunteered, provided informed consent, and completed the data collection forms. Nurses working exclusively in neonatal or pediatric intensive care, nurses with less than 1 year of adult intensive care experience, non-nurse health professionals, and forms that were incomplete or not usable were excluded. Complete eligible data from 198 nurses were analyzed, corresponding to 88.0% of the accessible workforce. Separate reason-specific counts for ineligibility, non-contact, refusal, and unusable forms were not retained; this reporting limitation is acknowledged below.

## Participants

The sample-size calculation was revised to correspond to the final multivariable analysis. For a multiple linear regression model containing 15 predictor parameters, a medium effect size (Cohen f^2^ = 0.15), a two-sided α = 0.05, and 95% power required 199 participants using the noncentral F distribution (Cohen, 1988). The achieved sample of 198 provided an estimated power of 94.9% under the same assumptions. This calculation concerns the overall regression model and does not guarantee adequate precision for sparse categories, such as self-reported psychiatric disorder.

### Data collection

Data were collected between January 2025 and April 2025. The researcher visited adult ICUs, informed ICU nurses who agreed to participate about the aim, importance, duration of the study, and confidentiality of the data, and obtained written and verbal consent. The estimated time to complete the surveys was 15–20 min. The data collection tools consisted of three parts: a sociodemographic information form, the Thanatophobia Scale (TS), and the Spirituality and Spiritual Care Rating Scale (SSCRS). Forms were checked for completeness before analysis.

## Sociodemographic information form

A 13-item form was developed by the researchers based on the literature (Arab et al., 2019; Baysal et al., 2024; Çamlıca et al., 2024; Sharif Nia et al., 2020). Yıldız Çifçioğlu and Harmancı Seren (2024) The form included questions on age, sex, marital status, number of children, educational status, years of professional experience, satisfaction with the current unit, presence of a serious physical illness, presence of a psychiatric disorder, experience of bereavement, the average monthly number of deaths in the unit, and the extent to which nurses were affected by the deaths they witnessed. The dataset recorded male and female categories; accordingly, the term self-reported sex is used consistently. Religious affiliation, religiosity, personal spirituality, spiritual-care education, social desirability, psychological resilience, burnout, and unit-level staffing were not measured.

## Thanatophobia Scale (TS)

The Thanatophobia Scale is a 7-point Likert-type instrument developed by Merrill et al. (1998) to assess death fear among healthcare professionals (Merrill et al., 1998). The Turkish validity and reliability study was conducted by Yıldız Çifçioğlu and Harmancı Seren (2024). The scale is unidimensional and consists of seven items. Responses are rated from 1 (“strongly disagree”) to 7 (“strongly agree”), yielding a total score between 7 and 49, with higher scores indicating higher levels of death fear. No validated low, moderate, or high cutoffs were applied, and the score was analyzed continuously. No item wording or response option was modified. In the current data, Cronbach’s α was 0.871 (bootstrap 95% CI [0.836, 0.897]). The Cronbach’s alpha coefficient was 0.87 in the original study (Merrill et al., 1998), 0.854 in the Turkish adaptation, and 0.87 in the present study, indicating high internal consistency (Büyüköztürk, 2018).

## Spirituality and Spiritual Care Rating Scale (SSCRS)

The SSCRS is a five-point Likert-type scale developed by McSherry et al. (2002) to assess perceptions of spirituality and spiritual care. The Turkish validity and reliability study was conducted by Ergül and Bayık (2007). The scale consists of 17 items rated from 1 (“strongly disagree”) to 5 (“strongly agree”). Four items (3, 4, 13, and 16) are reverse scored. Total scores range from 17 to 85, with higher scores indicating higher levels of perceived spirituality and spiritual care. The item mean (total divided by 17) was also reported to facilitate interpretation. Because the scale has no validated diagnostic or categorical thresholds, scores were not classified as low, moderate, or high. The scale does not directly measure observed care perception, frequency of spiritual-care delivery, or competence. In the current data, Cronbach’s α was 0.860 (bootstrap 95% CI [0.827, 0.884]). The Cronbach’s alpha coefficient was 0.64 in the original study by McSherry et al., 0.76 in the Turkish adaptation, and 0.843 in the present study. These values indicate a high level of internal consistency (Büyüköztürk, 2018).

### Data preparation and statistical analysis

Analyses were performed with IBM SPSS Statistics for Windows, Version 22.0, and independently verified in Python 3.13.5 using pandas, NumPy, SciPy, and statsmodels. Data quality checks included duplicated identifiers, missing item responses, values outside the theoretical response ranges, scale-scoring formulas, and consistency between stored and recalculated totals. No item-level missing values or out-of-range scale responses were identified; consequently, no imputation was performed. TS scores were calculated as the sum of all seven items. SSCRS scores were recalculated from all 17 items after reverse scoring items 3, 4, 13, and 16.

Categorical variables were summarized as *n* (%), and continuous variables as mean ± standard deviation, median (interquartile range), and range when relevant. Scale distributions were assessed using histograms, Q-Q plots, skewness, kurtosis, and residual diagnostics. Internal consistency was estimated with Cronbach’s α and 5,000-resample nonparametric bootstrap confidence intervals.

The primary bivariate association between TS and SSCRS scores was evaluated with Pearson correlation and a Fisher z 95% confidence interval. Spearman’s rank correlation was used as a sensitivity analysis. Exploratory comparisons of TS and SSCRS across two-category variables used Welch’s t test and Hedges g; variables with three or more categories used Welch’s analysis of variance and η^2^. To limit multiplicity, Benjamini-Hochberg false discovery rate (FDR) adjustment was applied jointly to the 20 categorical comparisons. Significant omnibus comparisons were followed by Welch pairwise tests with Holm adjustment. Correlations with professional experience, intensive care experience, and number of deaths witnessed per month were also FDR adjusted.

Hierarchical multiple linear regression was used with SSCRS total score as the outcome. Block 1 included age group, self-reported sex, education, professional experience, intensive care experience, deaths witnessed per month, intensive care unit satisfaction, bereavement, serious physical illness, and psychiatric disorder. Block 2 added emotional response to witnessed death, and Block 3 added TS score. Predictor selection was theory-informed rather than based solely on univariable *p*-values. Unstandardized coefficients, HC3 robust standard errors, 95% confidence intervals, *p*-values, R^2^, adjusted R^2^, and change in R^2^ were reported. Linearity, model specification, residual distribution, homoscedasticity, independence, multicollinearity, and influential observations were assessed with residual plots, the Ramsey RESET test, Shapiro-Wilk test, Breusch-Pagan test, Durbin-Watson statistic, variance inflation factors, externally studentized residuals, leverage, and Cook’s distance. Robust M-estimation was used as an additional sensitivity analysis. Statistical significance was set at two-sided *p* < 0.05; FDR-adjusted *q* < 0.05 was used for exploratory multiple comparisons.

## Ethical considerations

Ethical approval was obtained from the Scientific Research Ethics Committee of the Graduate Education Institute of Canakkale Onsekiz Mart University (Decision date:31.03.2023; No:2023-YÖNP-0271). Written institutional permission was obtained from Canakkale Provincial Health Directorate (02.10.2024 date and E-97769597-799-255606077 no) and the Canakkale Onsekiz Mart University Hospital (28.10.2024 date and E27222899-622.02-2400281095 no). Permission to use the scales was obtained via e-mail from the relevant authors. The aim of the study was explained to nurses who agreed to participate, and their verbal and written informed consent was obtained. All stages of the research were conducted in accordance with the Declaration of Helsinki.

## Results

### Participant characteristics

The analytic sample comprised 198 intensive care nurses. Most participants were female (77.3%), married (66.2%), and bachelor’s degree graduates (61.6%); 50.5% were aged 31–45 years. Mean professional experience was 12.79 ± 8.04 years, mean intensive care experience was 8.47 ± 5.09 years, and the mean number of deaths witnessed per month was 6.22 ± 3.81. Most participants were satisfied with their intensive care unit (83.3%), and 61.6% had experienced the loss of a significant person. Regarding witnessed deaths, 7.6% reported being affected, 24.7% reported not being affected, and 67.7% reported that their response varied according to the patient’s condition (Table 1).

**TABLE 1:** Participant characteristics (*N* = 198).

Characteristic	Category	*n* (%)
Age (years)	23–30	73 (36.9)
	31–45	100 (50.5)
	46–60	25 (12.6)
Self-reported sex	Male	45 (22.7)
	Female	153 (77.3)
Marital status	Married	131 (66.2)
	Single	67 (33.8)
Number of children	None	97 (49.0)
	1	44 (22.2)
	≥ 2	57 (28.8)
Serious physical illness	Yes	14 (7.1)
	No	184 (92.9)
Psychiatric disorder	Yes	10 (5.1)
	No	188 (94.9)
Education	Associate degree	36 (18.2)
	Bachelor’s degree	122 (61.6)
	Postgraduate	40 (20.2)
Professional experience	1–5 years	38 (19.2)
	6–10 years	66 (33.3)
	11–15 years	34 (17.2)
	16–20 years	27 (13.6)
	≥ 21 years	33 (16.7)
Intensive care experience	1–5 years	71 (35.9)
	6–10 years	77 (38.9)
	≥ 11 years	50 (25.3)
Satisfied with intensive care unit	Yes	165 (83.3)
	No	33 (16.7)
Loss of a significant person	Yes	122 (61.6)
	No	76 (38.4)
Deaths witnessed/month	0–5	119 (60.1)
	6–10	54 (27.3)
	≥ 11	25 (12.6)
Emotional response to witnessed death	Affected	15 (7.6)
	Not affected	49 (24.7)
	Varied with patient condition	134 (67.7)

Exact continuous values: professional experience 12.79 ± 8.04 years; intensive care experience 8.47 ± 5.09 years; deaths witnessed/month 6.22 ± 3.81.

### Scale distributions and internal consistency

All TS and SSCRS item responses were present and within the theoretical response ranges. The TS mean was 26.52 ± 9.02 (range 7–49), and the SSCRS mean was 59.95 ± 9.90 (range 31–81). The SSCRS item mean was 3.53 ± 0.58 on the 1–5 response metric. Neither scale was categorized as low, moderate, or high because validated cutoffs were unavailable. Skewness and kurtosis values were close to zero, and internal consistency was good for both scales (Table 2).

**TABLE 2:** Descriptive statistics and internal consistency of the study measures.

Measure	Possible range	Observed range	Mean	SD	Skewness	Kurtosis	Cronbach α	95% CI
TS total	7–49	7–49	26.52	9.02	0.088	−0.493	0.871	[0.836, 0.897]
SSCRS total	17–85	31–81	59.95	9.90	−0.195	0.027	0.860	[0.827, 0.884]
SSCRS item mean	1–5	1.82–4.76	3.53	0.58	–	–	–	–

CI, confidence interval; SSCRS, Spirituality and Spiritual Care Rating Scale; TS, Thanatophobia Scale. Cronbach α confidence intervals were based on 5,000 bootstrap resamples. SSCRS items 3, 4, 13, and 16 were reverse scored before all 17 items were summed.

### Exploratory demographic and occupational associations

After FDR adjustment, female nurses had higher TS and SSCRS scores than male nurses (TS: *q* < 0.001, Hedges *g* = −0.635 for male minus female; SSCRS: *q* < 0.001, *g* = −0.887). Nurses who had not experienced the loss of a significant person had higher TS scores than those who had experienced such a loss (*q* = 0.036, *g* = −0.379 for bereavement yes minus no). Emotional response to witnessed death was associated with both TS (*q* < 0.001, η^2^ = 0.163) and SSCRS (*q* < 0.001, η^2^ = 0.196). For TS, all three emotional-response groups differed in Holm-adjusted pairwise comparisons: affected > varied with patient condition > not affected. For SSCRS, the affected and varied groups each scored higher than the not-affected group, whereas the affected and varied groups did not differ. Other categorical comparisons did not remain statistically significant after FDR adjustment (Supplementary Tables 1, 2).

The number of deaths witnessed per month was inversely correlated with both TS (*r* = −0.246, 95% CI [−0.372, -0.110], *q* = 0.002) and SSCRS (*r* = −0.244, 95% CI [−0.370, -0.108], *q* = 0.002). Professional experience and intensive care experience were not significantly correlated with either scale after FDR adjustment (Table 3). These exploratory associations should not be interpreted causally.

**TABLE 3:** Correlations of study measures and continuous occupational variables.

Variables	r	95% CI	*p*	q	Spearman ρ	*p*
TS with SSCRS	0.299	[0.166, 0.421]	< 0.001	–	0.301	< 0.001
Professional experience (years) with TS	0.056	[−0.084, 0.194]	0.432	0.648	0.032	0.651
Professional experience (years) with SSCRS	0.082	[−0.058, 0.219]	0.251	0.501	0.053	0.457
ICU experience (years) with TS	0.003	[−0.137, 0.142]	0.971	0.971	−0.025	0.732
ICU experience (years) with SSCRS	0.021	[−0.119, 0.160]	0.771	0.925	−0.040	0.578
Deaths witnessed/month with TS	−0.246	[−0.372, −0.110]	< 0.001	0.002	−0.221	0.002
Deaths witnessed/month with SSCRS	−0.244	[−0.370, −0.108]	< 0.001	0.002	−0.292	< 0.001

CI, confidence interval; SSCRS, Spirituality and Spiritual Care Rating Scale; TS, Thanatophobia Scale. *q* values are Benjamini-Hochberg FDR-adjusted across the six occupational correlations. The TS–SSCRS association was the prespecified primary bivariate analysis and was not included in that FDR family.

### Association between thanatophobia and SSCRS scores

TS and SSCRS scores showed a positive, modest bivariate association (Pearson *r* = 0.299, 95% CI [0.166, 0.421], *p* < 0.001). The sensitivity analysis was nearly identical (Spearman ρ = 0.301, *p* < 0.001). In the simple linear model, a one-point higher TS score was associated with a 0.328-point higher SSCRS score (*B* = 0.328, HC3 95% CI [0.146, 0.511], *p* < 0.001); R^2^ = 0.089 and adjusted R^2^ = 0.085. Thus, the unadjusted model accounted for 8.9% of SSCRS variance, representing a modest explanatory contribution rather than a strong or causal effect.

### Multivariable analysis

The hierarchical model results are presented in Table 4. Demographic, occupational, health, bereavement, and death-frequency covariates in Block 1 explained 20.2% of SSCRS variance. Adding emotional response to witnessed death increased R^2^ by 0.071 (*p* < 0.001). Adding TS in the final block increased R^2^ by 0.009, but this increment was not statistically significant (*p* = 0.124). The full model was significant, *F*(15,182) = 4.77, *p* < 0.001, with R^2^ = 0.282 and adjusted R^2^ = 0.223.

**TABLE 4:** Hierarchical regression model summary for SSCRS total score.

Model	Predictors added	R^2^	Adjusted R^2^	Δ R^2^	F change	df	*p*
1	Demographic, occupational, health, bereavement, and death-frequency covariates	0.202	0.150	0.202	–	12, 185	< 0.001
2	Emotional response to witnessed death	0.273	0.217	0.071	8.95	2, 183	< 0.001
3	Thanatophobia Scale total score	0.282	0.223	0.009	2.39	1, 182	0.124

SSCRS, Spirituality and Spiritual Care Rating Scale. Model 1 included age group, self-reported sex, education, professional experience, intensive care experience, deaths witnessed/month, intensive care unit satisfaction, bereavement, serious physical illness, and psychiatric disorder. The full model was *F*(15,182) = 4.77, *p* < 0.001.

In the fully adjusted model with HC3 standard errors, female sex was associated with a 4.91-point higher SSCRS score than male sex (95% CI [1.42, 8.40], *p* = 0.006). Compared with nurses who reported not being affected by witnessed deaths, those who reported being affected had scores 8.66 points higher (95% CI [3.26, 14.06], *p* = 0.002), and those whose responses varied with the patient’s condition had scores 6.59 points higher (95% CI [2.36, 10.81], *p* = 0.002). TS was not independently associated with SSCRS score after adjustment (*B* = 0.122, 95% CI [−0.078, 0.321], *p* = 0.231; Table 5).

**TABLE 5:** Fully adjusted regression model for SSCRS total score.

Predictor	B	HC3 SE	t	*p*	95% CI
Intercept	48.129	4.064	11.84	< 0.001	[40.110, 56.148]
Age 31–45 vs. 23–30	−0.913	1.934	−0.47	0.637	[−4.729, 2.903]
Age 46–60 vs. 23–30	−3.065	4.610	−0.66	0.507	[−12.160, 6.030]
Female vs. male	4.910	1.767	2.78	0.006	[1.423, 8.396]
Bachelor’s vs. associate degree	2.662	1.572	1.69	0.092	[−0.438, 5.763]
Postgraduate vs. associate degree	3.184	2.419	1.32	0.190	[−1.589, 7.958]
Not satisfied vs. satisfied	−1.597	2.133	−0.75	0.455	[−5.806, 2.612]
No bereavement vs. bereavement	−0.605	1.411	−0.43	0.669	[−3.390, 2.180]
Physical illness: yes vs. no	−0.735	2.284	−0.32	0.748	[−5.241, 3.771]
Psychiatric disorder: yes vs. no	−3.246	2.738	−1.19	0.237	[−8.648, 2.156]
Affected vs not affected	8.663	2.736	3.17	0.002	[3.264, 14.061]
Varies vs. not affected	6.587	2.141	3.08	0.002	[2.364, 10.811]
Professional experience (years)	0.070	0.202	0.35	0.727	[−0.327, 0.468]
ICU experience (years)	−0.065	0.198	−0.33	0.742	[−0.457, 0.326]
Deaths witnessed/month	−0.221	0.206	−1.08	0.283	[−0.627, 0.184]
Thanatophobia score	0.122	0.101	1.20	0.231	[−0.078, 0.321]

B, unstandardized coefficient; CI, confidence interval; HC3 SE, heteroscedasticity-consistent standard error; SSCRS, Spirituality and Spiritual Care Rating Scale. Reference categories were age 23–30 years, male sex, associate degree, satisfied with the intensive care unit, bereavement present, no serious physical illness, no psychiatric disorder, and not affected by witnessed death. R^2^ = 0.282; adjusted R^2^ = 0.223.

Diagnostics did not indicate material violation of the linear model: Durbin-Watson = 1.75; Breusch-Pagan *p* = 0.213; Shapiro-Wilk *p* = 0.777; Ramsey RESET *p* = 0.097; and maximum variance inflation factor = 4.65. One observation had an externally studentized residual above 3, although the maximum Cook’s distance was 0.087. HC3 inference was therefore retained. Robust M-estimation produced a similar TS coefficient (*B* = 0.134, 95% CI [−0.018, 0.286], *p* = 0.085), supporting the conclusion that the adjusted TS association was not statistically significant.

## Discussion

This study was conducted to determine the relationship between thanatophobia and spiritual care perception among intensive care nurses. In this study, the participants’ mean TS score was high (26.52 ± 9.02). In Türkiye, several studies examined nurses’ fear of death. In the Turkish validity and reliability study of the TS conducted by Yıldız Çifçioğlu and Harmancı Seren (2024), nurses’ TS scores were found to be high (4.39 ± 1.38). Another study with ICU nurses in a different university hospital reported a high mean TS score (30.17 ± 9.37) and indicated that nurses who had lost a significant person or experienced difficulties while caring for terminally ill patients had higher levels of fear of death compared with other nurses (Çamlıca et al., 2024). In an online study conducted with nurses in Türkiye during the pandemic, the TS mean score was at a moderate level (5.22 ± 1.57) (Şanlıtürk and Erdoğan, 2022). Other studies used different instruments to assess death fear. In a study including all nurses working in a university hospital, the mean Thorson–Powell Death Anxiety Scale score was moderate (48.65 ± 14.92) (Üstükuş and Eskimez, 2021). Among emergency nurses working in four hospitals in eastern Türkiye, the mean TS score was high (35.37 ± 10.49) (Kartal et al., 2024). In another study conducted with nurses working in four hospitals in one province using the Frommelt Attitudes Toward Care of the Dying Scale (FATCOD), the mean score was found to be high (77.98 ± 8.81) (Seven and Sert, 2020). Findings from the international literature are also consistent regarding nurses’ fear of death. In a study with ICU nurses in Iran, the mean score on the Death Anxiety Scale–Extended (DAS-E) was high (148.63 ± 31.40) Karkhah et al. (2024). In Jordan, a study with nurses having at least one year of experience reported high Collett–Lester Fear of Death Scale (CL-FODS) scores (96.93 ± 23.69) (Rababa et al., 2023). In Israel, nurses’ death anxiety scores were also high (4.15 ± 1.76) (Kagan, 2021). In Oman, the mean death anxiety score among ICU nurses was moderate (38.23 ± 6.96) (Qutishat, 2025). In a study with hospice nurses in the southern United States, fear of death was relatively low (2.60 ± 1.10), and nurses who held a more positive view of death were reported to have lower fear of death (Barnett et al., 2021). These studies indicate a linear association between nurses’ level of fear of death and the difficulties experienced while caring for terminally ill patients, as well as avoidance behaviors toward dying patients. They also suggest that nurses’ internalization of these experiences as personal encounters may be a potential factor contributing to heightened fear of death (Karkhah et al., 2024; Chang and Lin, 2023; Barnett et al., 2021; Üstükuş and Eskimez, 2021; Norouzi et al., 2024).

Although levels of fear of death reported in previous studies vary, they are generally high, which is consistent with the findings of the present study. Nurses’ fear of death may be influenced by multiple factors, including age, professional experience, physical health, psychological resilience, spirituality, sociocultural values, social context, pandemics, work environment, patients’ condition, frequency of exposure to death, work-related stress, bereavement, communication with relatives of dying patients, social support, and emotional intelligence. High levels of thanatophobia in nurses may be associated with anxiety, work stress, burnout, avoidance of dying patients, psychological problems, reduced quality of life, limited social activities, difficulties in coping with thoughts of death, and decreased life and job satisfaction. While struggling mentally with fear of death, nurses may become reluctant to care for dying patients, consider leaving their job, and deliver lower-quality care (Chang and Lin, 2023).

In this study, the nurses’ SSCRS scores were high (52.79 ± 9.22). In Türkiye, several studies examined nurses’ spirituality and spiritual care. In a study with ICU nurses in a state hospital, SSCRS scores were high (62.86 ± 7.33) (Kutlu et al., 2020). Similarly, high SSCRS scores (52.03 ± 5.01) were reported among medical ward nurses in a university hospital in western Türkiye (Karaman et al., 2022). In a study conducted with nurses working in 10 provinces affected by the earthquake, the mean SSCRS score was high (49.49 ± 2.73) (Karaca and Aydın Özkan, 2025). Another study with ICU nurses in a state hospital also found high SSCRS scores (3.70 ± 0.38)(Meşe et al., 2025). International evidence is in line with these findings. In a study with neonatal intensive care nurses in Iran, SSCRS scores were high (67.44 ± 9.38), and spiritual care was reported not to be limited to religious practices (Sadeghi et al., 2025). Other studies used different instruments to evaluate nurses’ spiritual care. Abusafia et al. (2021) reported high Spiritual Care Competence Scale (SCCS) scores (95.44) among nurses in a public hospital in Malaysia (Abusafia et al., 2021). High SCCS scores were also found in studies involving nurses in Taiwan (96.14) (Chen et al., 2020), China (60.88) (Guo et al., 2021), Taiwan (3.14) (Hsieh et al., 2020), Iran (109.08) [24] (Heidari et al., 2025), and Türkiye (3.54 ± 3.52) (Karaman and Sağkal Midilli, 2022). Similarly, high SCCS scores were reported among nurses in multiple hospitals across China (3.68) (Li et al., 2022), in two regions of Poland (3.87) (Machul et al., 2022), and in a training and research hospital in Türkiye (3.88) (Özakar Akça et al., 2022). High scores were also observed among oncology nurses in Türkiye (103.81)(Semerci et al., 2021) and among nurses working in palliative care, oncology, and ICUs in a state hospital in Türkiye (Kurtgöz et al., 2024), as well as among nurses in five hospitals in Ethiopia (3.14) (Seid and Abdo, 2022). Another tool used to assess spiritual care practices is the Nurse Spiritual Care Therapeutics Scale (NSCTS). In Iran, Merati-Fashi et al. (2021) reported low NSCTS scores (27.32 ± 9.17) among nurses in hospitals across four provinces. In Malaysia, Atarhim et al. (2019) found high NSCTS scores (54.08 ± 13.82); in Taiwan, Chen et al. (2020) reported relatively low scores (30.51 ± 12.80) among graduate nurses. In Türkiye, Aslan et al. (2020), found relatively low NSCTS scores (38.43 ± 1.89) among nurses in a training and research hospital (Aslan et al., 2020), whereas Ricci-Allegra (2018) reported high scores (55.36 ± 1.87) among palliative care nurses in the United States with at least 1 year of experience. In a study from Türkiye, Kudubes et al. (2021) reported that as nurses’ fear of death increased, their willingness to provide spiritual care decreased, a finding contrary to that of the present study. This discrepancy may be attributable to the fact that the study by Kudubes et al. was conducted during the COVID-19 pandemic, a period characterized by profound uncertainty. In contrast, Rahman et al. (2021) reported high mean SSCRS scores (46.20 ± 5.22) among nurses in two provinces in Türkiye and found that nurses with higher fear of death had higher perceptions of spiritual care. Overall, although nurses’ perceptions of spiritual care vary across studies, they are generally high. This variation may be related to factors such as the instruments used, cultural and societal context, traditions, patient load, nursing shortages, and working conditions.

The high levels of spirituality and spiritual care perceptions among the nurses in this study indicate that they value spirituality, are sensitive to the topic, and have good awareness in this area. In Turkish society, the prominence of spirituality may be associated with the country’s predominantly Muslim population, deep-rooted faith traditions, the continuity of religious and cultural rituals, strong family ties, and adherence to communal values. The intergenerational transmission of values and the religious and sociocultural significance attributed to life events such as marriage, birth, illness, death, and funerals may contribute to higher levels of spirituality. In addition, undergraduate courses on human relations, ethics, and professional values may have a positive influence on nurses’ perceptions. However, high perceptions of spirituality and spiritual care do not necessarily indicate that nurses consistently provide spiritual care in practice. Organizational and environmental factors, including insufficient nurse staffing, lack of support personnel, high workload, overtime, lack of appropriate personnel (e.g., chaplains or spiritual care providers), and limited resources, may hinder the provision of spiritual care. Individual factors such as age, sex, sociocultural background, having received education on spirituality, religious belief or non-belief, psychological status, stress level, life expectations, sense of purpose, hope, personal interests (e.g., art, music, meditation, sports), and the broader social context may also affect nurses’ spiritual care practices. Furthermore, nurses and patients may differ in terms of religious affiliation, beliefs, and spiritual orientation. In such cases, it is essential that nurses provide care that respects, and responds to, the patient’s own spirituality, religion, and values. For critically ill patients, nurses may collaborate with the family and healthcare team to invite a spiritual care provider or religious official when needed. The key point is that nurses should recognize and assess patients’ spiritual needs and plan care accordingly. To achieve this, nurses must first hold an accurate understanding of spirituality and spiritual care. Nurse academics, particularly those specializing in spirituality and spiritual care, have made substantial contributions to the development of nursing knowledge in this field (Timmins et al., 2025). Education is essential for enhancing spiritual care; its outcomes support both nurses and patients in exploring meaning and purpose in life and in improving overall well-being (Alshakhshir et al., 2025).

## Limitations of the study

Several limitations should be considered. First, the cross-sectional design precludes temporal and causal inference; the terms effect and prediction in a longitudinal sense are not warranted. Second, all measures were self-reported and collected in the same survey session, creating potential common-method and social-desirability bias. Third, the SSCRS measures perceptions and understanding rather than observed spiritual-care behavior, practice frequency, or competence. Fourth, participants were volunteers from adult intensive care units in two public hospitals in one province; findings may not generalize to private hospitals, other regions, pediatric or neonatal units, palliative care, oncology, or non-nursing professionals.

Fifth, although 198 complete cases represented 88.0% of the accessible workforce, separate counts and reasons for ineligibility, non-contact, refusal, or unusable forms were not retained. Selection and nonresponse bias therefore cannot be quantified. Sixth, important constructs—including personal spirituality, religiosity, religious affiliation, spiritual-care education, coping style, resilience, empathy, burnout, moral distress, social desirability, workload, and unit-level characteristics—were not measured, leaving residual confounding. Seventh, physical illness and psychiatric disorder categories were sparse, reducing coefficient precision despite inclusion in the adjusted model. Eighth, emotional response to witnessed death and monthly death exposure were assessed with brief self-report items rather than validated multidimensional measures. Finally, the study may have been underpowered for very small incremental effects or interaction terms, and no interaction hypotheses were tested.

## Conclusion

Intensive care nurses’ thanatophobia was modestly and positively associated with their perceptions of spirituality and spiritual care at the bivariate level. However, thanatophobia did not show a statistically significant independent association after demographic, occupational, health, bereavement, death-exposure, and emotional-response variables were considered. Female sex and nurses’ reported emotional response to witnessed deaths were more consistent adjusted correlates of SSCRS scores. These findings support a multifactorial and noncausal interpretation. They do not demonstrate that fear of death increases spiritual care perceptions or that favorable perceptions correspond to actual spiritual care behavior.

## Implications for nursing management and research

Nurse managers and educators may consider offering evidence-informed opportunities for reflection, communication about death and dying, grief support, and education on assessing and responding to spiritual needs. Such initiatives should be presented as potentially useful strategies requiring evaluation, not as interventions proven effective by this study. Support should be voluntary, confidential, culturally sensitive, inclusive of religious and nonreligious perspectives, and linked to professional mental-health services when clinically indicated. Future research should use longitudinal or prospective designs, validated measures of death competence and spiritual-care behavior, and multilevel data on staffing and unit context. Qualitative interviews could clarify why some nurses report that witnessed deaths affect them and how this experience relates to meaning, coping, and spiritual care. Intervention studies are needed before recommending specific therapy rooms, support programs, or educational packages as effective outcomes of this evidence.

## Data Availability

The original contributions presented in this study are included in this article/Supplementary material, further inquiries can be directed to the corresponding author.

## References

[B1] Abusafia A. H. Mamat Z. Rasudin N. S. Bakar M. (2021). Spiritual care competence among Malaysian staff nurses. *Nurse Media J. Nursing* 11, 1–9. 10.14710/nmjn.v11i1.34757

[B2] Akpınar Y. Y. Dönmez ÇF. Aştı N. (2025). Experiences related to grief and death from the perspective of nurses caring for individuals approaching the end of life: A qualitative study. *J. Psychiatric Nursing/Hemşireleri Derneği* 16, 48–59. 10.14744/phd.2025.53911

[B3] Alshakhshir N. S. Ersig A. L. Ward E. Hendricks-Ferguson V. L. Montgomery K. E. (2025). An evolutionary concept analysis of spiritual competence in nursing. *J. Adv. Nursing* 81, 2977–2989. 10.1111/jan.16660 39618128 PMC12080088

[B4] Anshasi H. A. Fawaz M. Aljawarneh Y. M. Alkhawaldeh J. F. M. (2024). Exploring nurses’ experiences of providing spiritual care to cancer patients: A qualitative study. *BMC Nursing* 23:207. 10.1186/s12912-024-01830-2 38539194 PMC10967064

[B5] Arab M. Seyed Bagheri S. H. Sayadi A. Heydarpour N. (2019). Comparison of death anxiety, death obsession, and humor among nurses working in medical-surgical departments and intensive care units. *Arch. Neurosci.* 6:e86398. 10.5812/ans.86398

[B6] Aslan H. Aktürk Ü Erci B. (2020). Validity and reliability of the Turkish version of the nurse spiritual care therapeutics scale. *Palliat. Supportive Care* 18, 707–712. 10.1017/S1478951520000267 32390585

[B7] Atarhim M. A. Lee S. Copnell B. (2019). An exploratory study of spirituality and spiritual care among malaysian nurses. *J. Relig. Health* 58, 180–194. 10.1007/s10943-018-0624-0 29679189

[B8] Barnett M. D. Reed C. M. Adams C. M. (2021). Death attitudes, palliative care self-efficacy, and attitudes toward care of the dying among hospice nurses. *J. Clin. Psychol. Med. Settings* 28, 295–300. 10.1007/s10880-020-09714-8 32170511

[B9] Baysal E. Demirkol H. Erol A. Kaçmaz E. D. Duka B. Agolli B. et al. (2024). Nurses’ perceptions of spirituality and spiritual care and influencing factors in Türkiye, Italy, and Albania: A multicultural study. *Healthcare* 12:1391. 10.3390/healthcare12141391 39057534 PMC11275283

[B10] Becker G. S. (1973). A theory of marriage: Part I. *J. Polit. Econ.* 81, 813–846. 10.1086/260084 42701821 PMC13546084

[B11] Büyüköztürk Ş (2018). *Sosyal bilimler için veri analizi el kitabı.* Ankara: Pegem Akademi.

[B12] Çamlıca T. Özkan A. Kartal H. (2024). The relationship between intensive care nurses’ tanatophobia levels and the conscience stress and compassion fatigue. *J. Health Nursing Manag.* 11, 333–343. 10.54304/SHYD.2024.36744

[B13] Chang W. P. Lin Y. K. (2023). Influence of basic attributes and attitudes of nurses toward death on nurse turnover: A prospective study. *Intern. Nursing Rev.* 70, 476–484. 10.1111/inr.12781 35768904

[B14] Chen M. L. Chen Y. H. Lin L. C. Chuang L. L. (2020). Factors influencing the self-perceived competencies in spiritual care of nurses in the long-term care facilities. *J. Nursing Manag.* 28, 1286–1294. 10.1111/jonm.13080 32589763

[B15] Chen S. Wu H. Sun M. Wei Q. Zhang Q. (2023). Effects of shift work schedules, compensatory sleep, and work–family conflict on fatigue of shift-working nurses in Chinese intensive care units. *Nursing Crit. Care* 28, 948–956. 10.1111/nicc.12909 37078518

[B16] Cohen J. (1988). Set correlation and contingency tables. *Appl. Psychol. Measurement* 12, 425–434. 10.1177/014662168801200410

[B17] Dalcalı B. K. Durgun H. Can Ş (2021). Spiritual well-being and quality of life in patients who receive treatment in oncology units. *J. Inonu University Health Services Vocational School* 9, 536–553. 10.33715/INONUSAGLIK.812182

[B18] El-Ashry A. M. Eltyebani S. Elsayed S. M. Khedr M. A. El-Sayed M. M. Ghoneam M. A. et al. (2025). Soulful support: Exploring critical care nurses’ spiritual caregiving towards end-of-life scenario. *Nursing Crit. Care* 30:e70026. 10.1111/nicc.70026 40207429

[B19] Ergül Ş Bayık A. (2007). Maneviyat ve manevi bakım dereceleme ölçeği”nin Türkçe formunun geçerlilik ve güvenilirliği. *Ege Üniversitesi Hemşirelik Yüksekokulu Dergisi* 23, 75–87.

[B20] Greenberg J. Pyszczynski T. Solomon S. (1986). “The causes and consequences of a need for self-esteem: A terror management theory,” in *Public Self and Private Self*, ed. Baumeister R. F. (New York, NY: Springer New York), 189–212.

[B21] Greenberg J. Solomon S. Pyszczynski T. (1997). “Terror management theory of self-esteem and cultural worldviews: Empirical assessments and conceptual refinements,” in *Advances in Experimental Social Psychology*, Vol. 29, ed Zanna M. P. (Cambridge, MA: Academic Press), 61–139.

[B22] Guidelines for the Providing of Spiritual Support Services in Health Care Institutions. (2015). *Presidency of Religious Affairs, Directorate of Legal Guidance*. Available online at: http://hukukmusavirligi.diyanet.gov.tr (accessed May 21, 2025).

[B23] Guo Y. F. Cross W. M. Lam L. Plummer V. Wang X. X. Wang S. S. (2021). Association between psychological capital and spiritual care competencies of clinical nurses: A multicentre cross-sectional study. *J. Nursing Manag.* 29, 1713–1722. 10.1111/jonm.13303 33682206

[B24] Heidari A. Abbaspour A. Heidari M. (2025). Training well-rounded healthcare professionals through developing religious/spiritual competencies: a grounded theory study in Iran. *BMC Med. Educ.* 25:1138. 10.1186/s12909-025-07557-1 40751151 PMC12317613

[B25] Hosseini S. M. Naseri-Salahshour V. Farsi Z. Esmaeili M. Sajadi M. Maddah Z. et al. (2022). The effect of spirituality-oriented psychological counseling on the fear of death among patients undergoing chronic hemodialysis: A randomized controlled trial. *Eur. J. Integr. Med.* 49:102103. 10.1016/j.eujim.2022.102103

[B26] Hsieh S. I. Hsu L. L. Kao C. Y. Breckenridge-Sproat S. Lin H. L. Tai H. C. et al. (2020). Factors associated with spiritual care competencies in Taiwan’s clinical nurses: A descriptive correlational study. *J. Clin. Nurs.* 29, 1599–1613. 10.1111/jocn.15141 31855298

[B27] International Council of Nursing [ICN] (2021). *The ICN Code of Ethics for Nurses (Revised ed.).* Geneva: ICN.

[B28] International Council of Nursing [ICN] (2025). *Renewing the Definitions of “Nursing” and “A Nurse”.* Geneva: ICN.

[B29] Kagan M. (2021). Social support moderates the relationship between death anxiety and psychological distress among Israeli nurses. *Psychol. Rep.* 124, 1502–1514. 10.1177/0033294120945593 32731799

[B30] Karaca T. Aydın Özkan S. (2025). Moral sensitivity, spiritual care perception, and compassion fatigue of nurses caring for earthquake victims. *Intern. Nursing Rev.* 72:e13066. 10.1111/inr.13066 39533804 PMC12138233

[B31] Karacan Y. Akkuş Y. Bayram R. Budak S. Ünlü A. A. (2024). Do spiritual well-being and pain intensity predict physical or mental components of health-related quality of life in patients with multiple myeloma? *Pain Management Nursing* 25, e367–e374. 10.1016/j.pmn.2024.05.006 38834417

[B32] Karahan C. Ermin D. Aygin D. (2020). The relationship evaluation of intensive care nurses’ spiritual well-being and spiritual care perceptions. *Sakarya Univ. J. Holistic Health* 3, 130–142.

[B33] Karaman A. Sağkal Midilli T. (2022). The relationship between nurses’ perceptions and competency about spiritual care and influencing factors. *Perspect. Psychiatric Care* 58, 1021–1028. 10.1111/ppc.12894 34105169

[B34] Karaman E. Erkin Ö Göl Ý (2022). The relationship between spiritual care levels of Turkish nurses with the spiritual well-being of their patients: An exploratory study. *J. Relig. Health* 61, 1882–1893. 10.1007/s10943-021-01194-w 33544321

[B35] Karkhah S. Jafari A. Paryad E. Kazemnejad Leyli E. Ghazanfari M. J. Osuji J. et al. (2024). Death anxiety and related factors among Iranian critical care nurses: A multicenter cross-sectional study. *OMEGA—J. Death Dying* 88, 1153–1167. 10.1177/00302228211062368 34911401

[B36] Kartal M. Kapıkıran G. Karakaş N. (2024). The effect of emergency nurses’ psychological resilience on their thanatophobic behaviors: A cross-sectional study. *OMEGA—J. Death Dying* 89, 1273–1285. 10.1177/00302228221128156 36117453

[B37] Kudubes A. A. Akıl Z. K. Bektaş M. Bektaş Ý (2021). Nurses’ attitudes towards death and their effects on spirituality and spiritual care. *J. Relig. Health* 60, 153–161. 10.1007/s10943-019-00927-2 31598824

[B38] Kurtgöz A. Keten Edis E. Erarslan R. (2024). Spiritual care competencies and the frequency of spiritual care practices of nurses in Turkey. *J. Relig. Health* 63, 1747–1760. 10.1007/s10943-023-01884-7 37540306

[B39] Kutlu Ö Ermin C. Aygin D. (2020). The relationship evaluation of intensive care nurses spiritual well-being and spiritual care perceptions. *Sakarya Univ. J. Holistic Health* 3, 130–142. Available online at: https://dergipark.org.tr/en/download/article-file/1710315

[B40] Li Y. Zeng X. Chen M. Hu Y. Wu J. Lu W. et al. (2022). Association between spiritual care competency and perceived professional benefit among nurses: A cross-sectional study. *J. Nursing Manag.* 30, 3368–3377. 10.1111/jonm.13793 36064199

[B41] Machul M. van Leeuwen R. Ozga D. Jurek K. Boczkowska S. Dobrowolska B. (2022). The level of spiritual care competence of Polish nurses and the psychometric properties of the spiritual care competence scale (SCCS). *BMC Nursing* 21:106. 10.1186/s12912-022-00889-z 35524308 PMC9073815

[B42] McSherry W. Draper P. Kendrick D. (2002). The construct validity of a rating scale designed to assess spirituality and spiritual care. *Intern. J. Nursing Stud.* 39, 723–734. 10.1016/S0020-7489(02)00014-7 12231029

[B43] Medin ŞT. Hintistan S. Özoran Y. (2020). Determination of death anxiety in physicians and nurses working in internal clinics. *J. Hacettepe Univ. Faculty Nurs.* 7, 262–270. 10.31125/hunhemsire.834176

[B44] Merati-Fashi F. Khaledi-Paveh B. Mosafer H. Ebadi A. (2021). Validity and reliability of the Persian version of the Nurse Spiritual Care Therapeutics Scale (NSCTS). *BMC Palliative Care* 20:56. 10.1186/s12904-021-00750-1 33845815 PMC8041019

[B45] Merrill J. Lorimor R. Thornby J. Woods A. (1998). Caring for terminally ill persons: Comparative analysis of attitudes (thanatophobia) of practicing physicians, student nurses, and medical students. *Psychol. Rep.* 83, 123–128. 10.2466/pr0.1998.83.1.123 9775670

[B46] Meşe S. Sazak Y. Kalaycı M. (2025). End-of-life spiritual care from the perspective of intensive care nurses in Türkiye: An exploratory mixed study. *J. Relig. Health* 64, 4088–4110. 10.1007/s10943-025-02401-8 40773079

[B47] Norouzi M. Ghorbani Vajargah P. Falakdami A. Mollaei A. Takasi P. Ghazanfari M. J. et al. (2024). A systematic review of death anxiety and related factors among nurses. *OMEGA—J. Death Dying* 89, 1473–1491. 10.1177/00302228221095710 35500143

[B48] Özakar Akça S. Gülnar E. Özveren H. (2022). Spiritual care competence of nurses. *J. Continuing Educ. Nursing* 53, 225–231. 10.3928/00220124-20220407-05 35510924

[B49] Qutishat M. (2025). Relationship between death anxiety and resilience among critical care nurses in Oman. *Nursing Crit. Care* 30:e13177. 10.1111/nicc.13177 39363844

[B50] Rababa M. Masha’al D. Shahrour G. (2023). Association of ageism with death anxiety, self-esteem, interpersonal reactivity, and symbolic immortality among nurses. *OMEGA—J. Death Dying* 87, 231–245. 10.1177/00302228211019200 34038172

[B51] Rahman S. Elbi H. Çakmakcı Çetinkaya A. Altan S. Ozan E. Pirinçci E. (2021). Factors that predict the perception of spirituality and spiritual care of nurses working in high-risk units and the effect of death anxiety. *Perspect. Psychiatric Care* 57, 473–480. 10.1111/ppc.12651 33196114

[B52] Regulation Amending the Nursing Regulation. (2011). *Official Gazette of the Republic of Turkey (Issue: 27910)*. Available online at: https://www.resmigazete.gov.tr/eskiler/2011/04/20110419-5.html (accessed April 25, 2025).

[B53] Ricci-Allegra P. (2018). Spiritual perspective, mindfulness, and spiritual care practice of hospice and palliative nurses. *J. Hospice Palliative Nurs.* 20, 172–179. 10.1097/NJH.0000000000000426 30063571

[B54] Sadeghi N. Heidari H. Heidarzadeh M. Moghimian M. (2025). Evaluation of nurses’ perception of spirituality and spiritual care of parents in neonatal intensive care units in Iran: A national study. *BMC Nursing* 24:1019. 10.1186/s12912-025-03680-y 40764926 PMC12323236

[B55] Şanlıtürk D. Erdoğan Ç (2022). Examination of nurses’ COVID-19 phobia and thanatophobia levels as per the service unit type. *J. TOGU Health Sci.* 2, 225–237. Available online at: https://izlik.org/JA36WF79ZN

[B56] Seid K. Abdo A. (2022). Nurse’s spiritual care competence in Ethiopia: A multicenter cross-sectional study. *PLoS One* 17:e0265205. 10.1371/journal.pone.0265205 35271676 PMC8912899

[B57] Semerci N. Uysal N. Bağçıvan G. Doğan M. A. Kostak E. Tayaz E. et al. (2021). Oncology nurses’ spiritual care competence and perspective about spiritual care services. *Turk. J. Oncol.* 36. 10.5505/tjo.2021.2682

[B58] Seven A. Sert H. (2020). How the nurses’ attitude for dying patients and their knowledge about palliative care? *Bezmialem Sci.* 8, 250–257. 10.14235/bas.galenos.2019.3419

[B59] Sharif Nia H. Pahlevan Sharif S. Koocher G. P. Yaghoobzadeh A. Haghdoost A. A. Mar Win M. T. et al. (2020). Psychometric properties of the death anxiety scale-extended among patients with end-stage renal disease. *OMEGA—J. Death Dying* 80, 380–396. 10.1177/0030222817733220 28982272

[B60] Tan H. N. Yıldız M. (2022). The formation and definition of the concept of “spiritual well-being.”. *Relig. Stud.* 25, 447–476. 10.15745/da.1175149

[B61] Timmins F. Attard J. Dobrowolska B. Connolly M. Caldeira S. Parissopoulos S. et al. (2025). Trends in spirituality and spiritual care in nursing—A discursive paper. *J. Adv. Nursing* 82, 6845–6856. 10.1111/jan.70231 40965160 PMC13176731

[B62] Uçar Ö Çelik S. Karahan E. Altıntaş S. Yücel M. (2024). Exploring the relationship between spiritual care and patient advocacy of nurses from generations X, Y and Z working in intensive care clinics: A cross-sectional study. *Intens. Crit. Care Nursing* 84:103754. 10.1016/j.iccn.2024.103754 38917680

[B63] Umberger W. Wilson M. (2024). Translating the biopsychosocial-spiritual model into nursing practice. *Pain Manag. Nursing* 25, 1–3. 10.1016/j.pmn.2023.12.008 38212091

[B64] Üstükuş A. Eskimez Z. (2021). The effect of death anxiety in nurses on their approach to dying patients: A cross-sectional study. *Perspect. Psychiatric Care* 57, 1929–1936. 10.1111/ppc.12768 33755209

[B65] von elm E. Altman D. G. Egger M. Pocock S. J. Gøtzsche P. C. Vandenbroucke J. P. (2007). The Strengthening the Reporting of Observational Studies in Epidemiology (STROBE) statement: Guidelines for reporting observational studies. *Lancet* 370, 1453–1457. 10.7326/0003-4819-147-8-200710160-00010 18064739

[B66] Wang W. Yang J. Bai D. Lu X. Gong X. Cai M. et al. (2024). Nurses’ perceptions and competencies about spirituality and spiritual care: A systematic review and meta-analysis. *Nurse Educ. Today* 132:106006. 10.1016/j.nedt.2023.106006 37922766

[B67] Yalom I. D. (1980). *Existential Psychotherapy.* New York, NY: Basic Books.

[B68] Yalom I. D. (2008). *Staring at the Sun: Overcoming the Terror of Death.* Hoboken, NJ: Jossey-Bass.

[B69] Yıldız Çifçioğlu Z. Harmancı Seren A. K. (2024). The validity and reliability of the Thanatophobia Scale–Turkish form: A psychometric study among nurses. *OMEGA—J. Death Dying* 89, 718–728. 10.1177/00302228221082755 35343307

[B70] Zulfiqar N. Habib A. Hassan M. (2021). Thanatophobia and psychological distress among health care workers during COVID-19. *Global Soc. Sci. Rev.* 6, 435–444. 10.31703/gssr.2021(VI-II).43

