# Supplementary material for: The relationship between thanatophobia and perceptions of spirituality and spiritual care among intensive care nurses
Source: Front Psychol. 2026 Sep 23;17:1945404. doi: 10.3389/fpsyg.2026.1945404 (PMC13642669; doi:10.3389/fpsyg.2026.1945404)
Supplement: Supplementary file 1 [file Supplementary_file_1.docx]

# **SUPPLEMENTARY TABLES**

**Supplementary Table S1. Thanatophobia Scale scores across participant characteristics**

| **Characteristic** | **Category** | **n** | **Mean±SD** | **p (q)** | **Effect size** |
| --- | --- | --- | --- | --- | --- |
| Age (years) | 23–30 | 73 | 27.34±10.64 | .020 (.066) | η²=0.024 |
|  | 31–45 | 100 | 25.25±8.24 |  |  |
|  | 46–60 | 25 | 29.20±5.61 |  |  |
| Self-reported sex | Male | 45 | 22.22±8.10 | <.001 (<.001) | Hedges g=-0.635 |
|  | Female | 153 | 27.78±8.91 |  |  |
| Marital status | Married | 131 | 26.15±8.21 | .460 (.649) | Hedges g=-0.120 |
|  | Single | 67 | 27.24±10.45 |  |  |
| Number of children | None | 97 | 26.91±10.20 | .228 (.434) | η²=0.011 |
|  | 1 | 44 | 27.55±8.33 |  |  |
|  | ≥2 | 57 | 25.07±7.15 |  |  |
| Serious physical illness | Yes | 14 | 28.43±6.50 | .286 (.477) | Hedges g=0.227 |
|  | No | 184 | 26.38±9.18 |  |  |
| Psychiatric disorder | Yes | 10 | 27.90±11.48 | .702 (.739) | Hedges g=0.160 |
|  | No | 188 | 26.45±8.90 |  |  |
| Education | Associate degree | 36 | 25.72±8.21 | .552 (.668) | η²=0.007 |
|  | Bachelor’s degree | 122 | 26.30±8.97 |  |  |
|  | Postgraduate | 40 | 27.93±9.88 |  |  |
| Satisfied with ICU unit | Yes | 165 | 26.72±8.59 | .568 (.668) | Hedges g=0.129 |
|  | No | 33 | 25.55±11.02 |  |  |
| Loss of a significant person | Yes | 122 | 25.22±9.11 | .009 (.036) | Hedges g=-0.379 |
|  | No | 76 | 28.61±8.51 |  |  |
| Emotional response to witnessed death | Affected | 15 | 34.47±7.62 | <.001 (<.001) | η²=0.163 |
|  | Not affected | 49 | 21.00±7.40 |  |  |
|  | Varied with patient condition | 134 | 27.65±8.66 |  |  |

**Note.** Welch t tests were used for two-category variables and Welch ANOVA for variables with three or more categories. q values are Benjamini-Hochberg FDR-adjusted across the 20 exploratory categorical comparisons. Hedges g is oriented as the first category minus the second category; η² is reported for multigroup comparisons. For emotional response, Holm-adjusted pairwise tests showed affected > varied with patient condition > not affected.

**Supplementary Table S2. SSCRS scores across participant characteristics**

| **Characteristic** | **Category** | **n** | **Mean±SD** | **p (q)** | **Effect size** |
| --- | --- | --- | --- | --- | --- |
| Age (years) | 23–30 | 73 | 59.70±10.68 | .741 (.741) | η²=0.002 |
|  | 31–45 | 100 | 59.86±9.94 |  |  |
|  | 46–60 | 25 | 61.08±7.33 |  |  |
| Self-reported sex | Male | 45 | 53.56±9.47 | <.001 (<.001) | Hedges g=-0.887 |
|  | Female | 153 | 61.84±9.25 |  |  |
| Marital status | Married | 131 | 59.69±9.51 | .621 (.690) | Hedges g=-0.077 |
|  | Single | 67 | 60.46±10.70 |  |  |
| Number of children | None | 97 | 60.52±10.28 | .239 (.434) | η²=0.014 |
|  | 1 | 44 | 61.09±8.66 |  |  |
|  | ≥2 | 57 | 58.12±10.07 |  |  |
| Serious physical illness | Yes | 14 | 61.57±8.20 | .463 (.649) | Hedges g=0.175 |
|  | No | 184 | 59.83±10.03 |  |  |
| Psychiatric disorder | Yes | 10 | 57.80±9.67 | .487 (.649) | Hedges g=-0.228 |
|  | No | 188 | 60.07±9.93 |  |  |
| Education | Associate degree | 36 | 58.06±9.37 | .125 (.311) | η²=0.021 |
|  | Bachelor’s degree | 122 | 59.66±9.93 |  |  |
|  | Postgraduate | 40 | 62.58±10.01 |  |  |
| Satisfied with ICU unit | Yes | 165 | 60.67±9.50 | .047 (.134) | Hedges g=0.434 |
|  | No | 33 | 56.39±11.22 |  |  |
| Loss of a significant person | Yes | 122 | 59.23±10.32 | .181 (.401) | Hedges g=-0.190 |
|  | No | 76 | 61.12±9.15 |  |  |
| Emotional response to witnessed death | Affected | 15 | 65.00±6.13 | <.001 (<.001) | η²=0.196 |
|  | Not affected | 49 | 52.45±9.49 |  |  |
|  | Varied with patient condition | 134 | 62.13±8.97 |  |  |

**Note.** Welch t tests were used for two-category variables and Welch ANOVA for variables with three or more categories. q values are Benjamini-Hochberg FDR-adjusted across the 20 exploratory categorical comparisons. Hedges g is oriented as the first category minus the second category; η² is reported for multigroup comparisons. For emotional response, the affected and varied groups each scored higher than the not-affected group; affected and varied groups did not differ after Holm adjustment.
